# *RppM*, Encoding a Typical CC-NBS-LRR Protein, Confers Resistance to Southern Corn Rust in Maize

**DOI:** 10.3389/fpls.2022.951318

**Published:** 2022-07-12

**Authors:** Shuai Wang, Xiaqing Wang, Ruyang Zhang, Qian Liu, Xuan Sun, Jidong Wang, Yuandong Wang, Jinfeng Xing, Ya Liu, Yanxin Zhao, Zi Shi, Aiguo Su, Chunhui Li, Senlin Xiao, Yanyan Jiao, Zhiyong Li, Ronghuan Wang, Wei Song, Jiuran Zhao

**Affiliations:** Maize Research Institute, Beijing Academy of Agriculture and Forestry Sciences, Beijing Key Laboratory of Maize DNA Fingerprinting and Molecular Breeding, Beijing, China

**Keywords:** maize, *RppM*, Southern corn rust (SCR), resistance gene, Kompetitive allele-specific PCR markers, marker-assisted selection (MAS)

## Abstract

Southern corn rust (SCR) caused by *Puccinia polysora* Underw. poses a major threat to maize production worldwide. The utilization of host SCR-resistance genes and the cultivation of resistant cultivars are the most effective, economical strategies for controlling SCR. Here, we identified and cloned a new SCR resistance gene, *RppM*, from the elite maize inbred line Jing2416K. *RppM* was found to encode a typical CC-NBS-LRR protein localized in both the nucleus and cytoplasm. This gene was constitutively expressed at all developmental stages and in all tissues examined, with the strongest expression detected in leaves at the mature stage. A transcriptome analysis provided further evidence that multiple defense systems were initiated in Jing2416K, including pathogen-associated molecular pattern-triggered immunity and effector-triggered immunity, reinforcement of cell walls, accumulation of antimicrobial compounds, and activation of phytohormone signaling pathways. Finally, we developed functional Kompetitive allele-specific PCR markers for *RppM* using two conserved SNP sites and successfully applied these functional markers for the detection of *RppM* and the cultivation of resistant maize cultivars, demonstrating their great potential utility in maize breeding.

## Introduction

Maize (*Zea mays* L.), one of the most important cultivated crops, is widely grown as a major food, fuel, and feed source worldwide (Li et al., 2019). Southern corn rust (SCR) is a widely distributed airborne fungal disease caused by *Puccinia polysora* Underw. that causes substantial yield losses and deterioration of grain quality in maize production. SCR, which was first reported in western Africa in 1949, is very prevalent in tropical and subtropical regions, with more than 50% yield losses reported in northern China during outbreaks and epidemics (Rhind et al., 1952; Zhou et al., 2008; Dolezal et al., 2009; Brewbaker et al., 2011). More recently, SCR epidemics have gradually spread to high-latitude areas and have proven disastrous in maize-producing regions of the world owing to global climate change (Wang S. et al., 2019; Mueller et al., 2020). The exploitation of SCR resistance (*R*) genes and the development of *R* gene-harboring maize lines are the most effective strategies for controlling SCR.

Plants have developed elaborate defense systems to protect against various pathogens. Defense responses mediated by *R* genes are triggered when specific effectors from pathogens are recognized by R proteins (Dodds and Rathjen, 2010; Ma et al., 2015; Saur et al., 2021). These defense responses are usually accompanied by the hypersensitive response, which triggers rapid programmed cell death at infection sites to inhibit pathogen growth in host plant tissues (Heath, 2000; Wang et al., 2017; Bi et al., 2021). A large number of *R* genes from a wide range of plant species have been characterized and cloned. The largest class of *R* genes encode nucleotide-binding site and leucine-rich repeat (NBS-LRR) proteins (Liu et al., 2007). The NBS domain is probably involved in ATP or GTP binding and hydrolyzation, whereas the LRR domain is generally associated with protein–protein interactions (Collier and Moffett, 2009; Qi et al., 2012). Plant NBS-LRR proteins can be divided into two classes according to their *N*-terminal domains: TIR-NBS-LRR and non-TIR-NBS-LRR, the latter often containing a coiled-coil (CC) domain at the N-terminal (Meyers et al., 2003; Liu et al., 2007).

At least 19 SCR resistance genes, including *Rpp1*–*Rpp11* (Storey and Howland, 1967; Dolezal et al., 2009; Brewbaker et al., 2011), *RppQ* (Chen et al., 2004; Zhou et al., 2008), *RppP25* (Zhao et al., 2013), *RppC* (Yao et al., 2013), *RppS* (Wu et al., 2015), *RppS313* (Wang B. et al., 2019), *RppCML496* (Lv et al., 2020), and *RppM* (Wang et al., 2020), have been identified to date from a variety of maize germplasm resources. Most of these genes have been mapped to the short arm of maize chromosome 10, but only one has previously been cloned: *RppC*, which encodes an NLR-type protein and triggers defense responses upon recognizing the avirulence effector AvrRppC (Deng et al., 2022).

In the present study, we cloned a new SCR resistance gene, *RppM*, from the elite maize inbred line Jing2416K. The *RppM* gene was found to encode a typical CC-NBS-LRR protein localized in both the nucleus and cytoplasm. This gene was constitutively expressed at all developmental stages and in all tissues examined, with the strongest expression detected in leaves at the mature stage. Functional pathway enrichment analysis suggested that multiple defense systems were initiated in Jing2416K, including PAMP-triggered immunity (PTI), effector-triggered immunity (ETI), reinforcement of cell walls, accumulation of antimicrobial compounds, and activation of phytohormone signaling pathways. In addition, we successfully applied functional Kompetitive allele-specific PCR (KASP) markers for *RppM* for the detection of *RppM* and cultivation of resistant maize cultivars.

## Materials and Methods

### Plant Materials

A total of 533 maize lines were used in this study. Among them, the resistant line Jing2416K and universally susceptible lines Jing2416, Jing724, JingMC01, and Jing92, were evaluated for SCR response. Maize inbred line B104 was used as a receptor genotype for *RppM* transformation assays. A GWAS population of 527 inbred lines and F_2_ individuals of Jing2416, and Jing2416K were used for a genotypic assay of two *RppM*-specific markers. In addition, four BC_3_F_2_ population derived from a cross between Jing2416K (donor parent) and Jing92H, JingX005, Jing2416B92, and Jing2416C92 (recurrent parents) were used in MAS breeding.

### Evaluation of Southern Corn Rust Resistance

All plant materials were grown at the experimental station of the Beijing Academy of Agriculture and Forestry Sciences in Sanya, Hainan Province, China. In Sanya, SCR develops naturally and becomes more severe as plants mature, when warm temperatures and high relative humidity promote the development and spread of the fungus *Puccinia polysora*. One row of line Jing2416 was grown as a susceptible control per 20 rows of each plant material. All inbred lines, F_2_ individuals, BC_3_F_2_ plants, and corresponding parents were infected *via* natural inoculation. SCR resistance was recorded at the grain filling stage according to a five-point scale as described by Zhao et al. (2013). Using lines Jing2416K and Jing2416 as controls, plants were classified as resistant or susceptible.

### Overexpression Analysis

Two putative NBS-LRR genes, *ORF4* and *ORF6*, were predicted from the Jing2416K genomic sequence as candidate genes for *RppM* within the *RppM* locus. To construct the overexpression vector, the CDS of *ORF4* and *ORF6* were amplified from Jing2416K using primer pairs C65F/R and C67F/R (Supplementary Table 1), respectively. The two amplified products were inserted into the binary vector p1132 using an In-Fusion HD Cloning kit (Takara, Kusatsu, Shiga, Japan) to generate the transformation plasmid *35S:ORF4* and *35S:ORF6*. All constructs were verified by sequencing and subsequently introduced into maize inbred line B104 by *A. tumefaciens*-mediated transformation as described in Lee and Zhang (2014).

### Sequence Annotation and Protein Domain Prediction

The genome region of *RppM* in Jing2416K was sequenced by PCR, primer pairs designed using Primer5 were listed in Supplementary Table 1. The protein molecular weight of RppM was computed using the Expasy website^1^). Major functional domains and motifs of RppM were predicted using the programs Simple Modular Architecture Research Tools^2^, Paircoil2^3^, and MEME^4^. Homologous sequences of *RppM* were identified using NCBI Blastp^5^ and Phytozome^6^. A phylogenetic tree was generated by the neighbor-joining method in MEGA v7 (Kumar et al., 2016).

### Quantitative Real-Time PCR Analysis

For expression pattern analysis of *RppM*, total RNA was extracted from roots, leaves, coleoptiles, and mesocotyls at the seedling stage and roots, first internodes, leaf sheaths, tassels, and ears at the mature stage using an RNAprep Pure Plant kit (Tiangen Biotech, Beijing, China). For validation of transcriptome data, total RNA was extracted from ear leaves of Jing2416K and Jing2416 at 48 and 62 DAS. First-strand cDNA was synthesized using a SuperScript II First-Strand cDNA Synthesis kit (Takara, Kusatsu, Shiga, Japan). Primer pairs designed using an online primer design tool^7^ are listed in Supplementary Table 1. qRT-PCR amplifications were performed using a SYBR Premix Ex *Taq* kit (Takara, Kusatsu, Shiga, Japan) as previously described (Wang et al., 2017), with the maize *Actin* gene (*Zm00001d012277*) used as an internal reference. Relative expression levels were determined by the 2^–^*^ΔΔCt^* method (Livak and Schmittgen, 2001).

### Subcellular Localization Analysis

A 3,126-bp fragment of the CDS of *RppM* was amplified with primers C65-GFP-F/R (Supplementary Table 1) and fused into the N-terminus of the GFP coding region in pM999-GFP vector to generate RppM-GFP fusion expression vector under the control of the CaMV 35S promoter using an In-Fusion HD Cloning kit. The fusion construct RppM-GFP was transformed into maize protoplast cells along with the nucleus marker D53-mCherry (Zhou et al., 2015) using a plant protoplast preparation and transformation kit (ZhongkeRuitai Biotechnology, Beijing, China). Green and red fluorescence was detected using a laser confocal scanning microscope (Leica TCS SP5).

### Transcriptome Sequencing and Analysis

Leaf samples of Jing2416 and Jing2416K were collected at 48 and 62 DAS, respectively, with each collection including three replicates. A total of 12 samples (2 materials × 2 time periods × 3 biological replicates) were subjected to transcriptome analysis. RNA extraction was carried out with a RNAprep Pure Plant kit. Sequencing libraries were generated using an Illumina Truseq RNA Sample Prep Kit for Illumina (Illumina, San Diego, CA, United States) and sequenced using on an Illumina Novaseq 6000 system, which mainly focused on small fragments of approximately 300 bp for sequencing (Modi et al., 2021). Generated high-quality clean reads were mapped to the maize reference genome (B73 RefGen_v4) using TopHat2 software (Kim et al., 2013). The mapped reads were assembled and spliced using Cufflinks software to obtain annotations of new transcripts (Trapnell et al., 2010). Gene expression levels were calculated as transcripts per million using RSEM software (Li and Dewey, 2011). Differential gene analysis between groups was performed using the thresholds of | log_2_FC| ≥1 and adjusted *p* ≤ 0.05 in edgeR (Robinson et al., 2010). Functional annotations of all transcripts and their corresponding genes were carried out using NR^8^, Swiss-Prot^9^, Pfam^10^, Clusters of Orthologous Groups of proteins^11^, Gene Ontology^12^, and Kyoto Encyclopedia of Genes and Genomes^13^ databases.

### Development of Kompetitive Allele-Specific PCR Markers

To develop KASP markers for *RppM*, we identified conserved SNPs by comparing CDSs of *RppM* alleles among resistant and susceptible inbred lines. KASP markers for conserved SNPs were designed using BatchPrimer3^14^ (Leal-Bertioli et al., 2015). KASP primer pairs, consisting of forward (Primer_AlleleFAM and Primer_AlleleHEX) and reverse (Primer_Common) primers (Supplementary Table 2), were synthesized by LGC Genomics (Hoddesdon, United Kingdom). Kompetitive allele-specific PCR reactions were performed in 1,536-well microplates in 1-μl reaction volumes containing approximately 30 ng of lyophilized DNA, 0.5 μl of KASP 2 × Master Mix (KBS-1016-011, LGC Genomics), 0.014 μl primer mixture, and 0.486 μl distilled deionized water. The PCR amplification protocol was as follows: 94°C for 15 min, followed by 10 cycles of 94°C for 20 s and touchdown from 61°C to 55°C for 1 min, and then 32 cycles of 94°C for 20 s and 55°C for 1 min. The fluorescence signals of PCR products were scanned using Pherastar, and the genotyping results were visualized and exported using Kraken software (LGC Genomics).

## Results

### Overexpression Analysis of *RppM*

**FIGURE 1:**
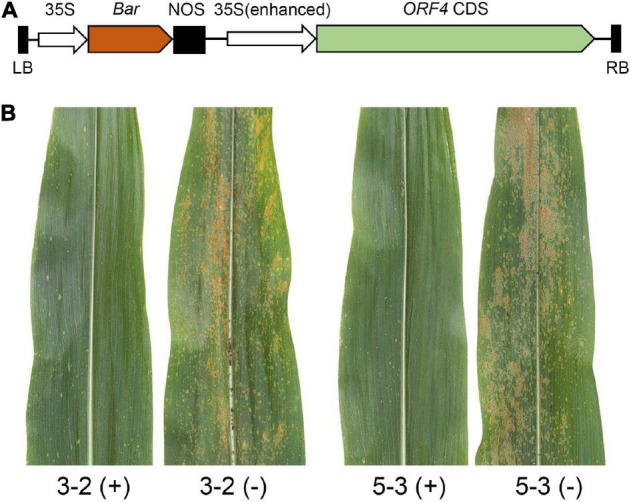
Overexpression analysis of *ORF4*. **(A)** Structure of the *ORF4* expression cassette used for Overexpression analysis. The brown arrow presents the *Bar* gene. LB, left border; RB, right border. **(B)** Phenotypes of two independent T_1_ overexpression lines (3-2 and 5-3) of *ORF4*.

We previously fine mapped *RppM* to a region spanning 110 kb on chromosome arm 10S and identified two genes (*ORF4* and *ORF6*) encoding a CC-NBS-LRR protein as potential *RppM* candidates (Wang et al., 2020). To determine whether either of the two candidate genes function as *RppM*, we separately introduced the coding DNA sequence (CDS) of *ORF4* and *ORF6* from Jing2416K under the control of 35S promoter into susceptible maize inbred line B104 via *Agrobacterium tumefaciens*-mediated transformation. Two independent T_1_ lines of *ORF4* or *ORF6* transgenic plants were obtained and then evaluated for SCR resistance by natural inoculation over 2 years (2020 and 2021) in Hainan Province. We found that the independent transgenic lines carrying *ORF6* were still highly susceptible to SCR (Supplementary Figure 1), which indicated that *ORF6* was neither responsible nor sufficient for *RppM*-mediated SCR resistance. In contrast, independent transgenic lines carrying *ORF4* exhibited stronger resistance to SCR compared with non-transgenic plants (Figure 1). Taken together, these results confirm that *ORF4* from Jing2416K is the functional *RppM* gene.

### Encoded Product of *RppM*: A Typical CC-NBS-LRR Protein

**FIGURE 2:**
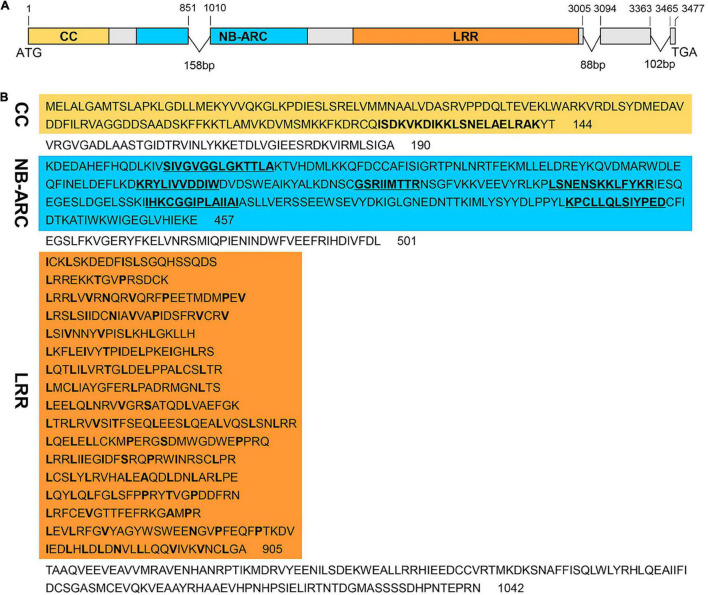
Structure of *RppM* and its predicted protein domain and motifs. **(A)** Gene structure of *RppM*. The boxes represent exons, and lines denote introns. ATG and TGA are the translation start and stop codons, respectively. Yellow, blue, and orange regions represent the coiled-coil (CC) coding region, nucleotide-binding site (NBS), and the leucine-rich repeat (LRR) domain, respectively. **(B)** The predicted domain and motifs of RppM. The CC motif is indicated in bold font in the yellow region. The NBS domain is shown in blue, and the six conserved motifs (P-loop, Kinase2, RNBS-B, RNBS-C, GLPL, and MHDV) of the NBS are indicated in bold font. The C-terminal LRR domain, shown in orange, contains 17 LRR motifs.

Sequence comparison between genomic DNA and the CDS revealed that *RppM* is composed of four exons separated by three introns (Figure 2A). The CDS of *RppM* consists of 3,126 nucleotides and encodes a putative protein comprising 1,042 amino acids with a molecular mass of 120 kDa. Protein motif analysis indicated that the N-terminus of the protein contains a potential CC domain (residues 122–142), an NBS domain containing six conserved motifs—P-loop (residues 206–219), Kinase2 (residues 283–293), RNBS-B (residues 311–319), RNBS-C (residues 336–348), GLPL (residues 366–379), and MHDV (residues 422–434)—and a C-terminal region containing 17 irregular LRR repeats (residues 502–905) (Figure 2B). Comparison of the amino acid sequence of *RppM* with those of orthologs from other organisms showed that the three domains and the main active amino acid sites are highly conserved in all aligned CC-NBS-LRR proteins (Supplementary Figure 2). *RppM* thus encodes a typical CC-NBS-LRR protein. According to a phylogenetic analysis of RppM and other CC-NBS-LRR-type proteins, i.e., RPM1, RPS2, RPP13, Pita, Pib, Pi25, RP1, Lr10, Pm3b, Mla1, Sw5, I2, R3a, R1, Bs2, Rpg1-b, and Fom-2, RppM is most similar to the barley powdery mildew resistant protein Lr10 (Supplementary Figure 3).

### Expression Pattern Analysis of *RppM* and Subcellular Localization of the RppM Protein

**FIGURE 3:**
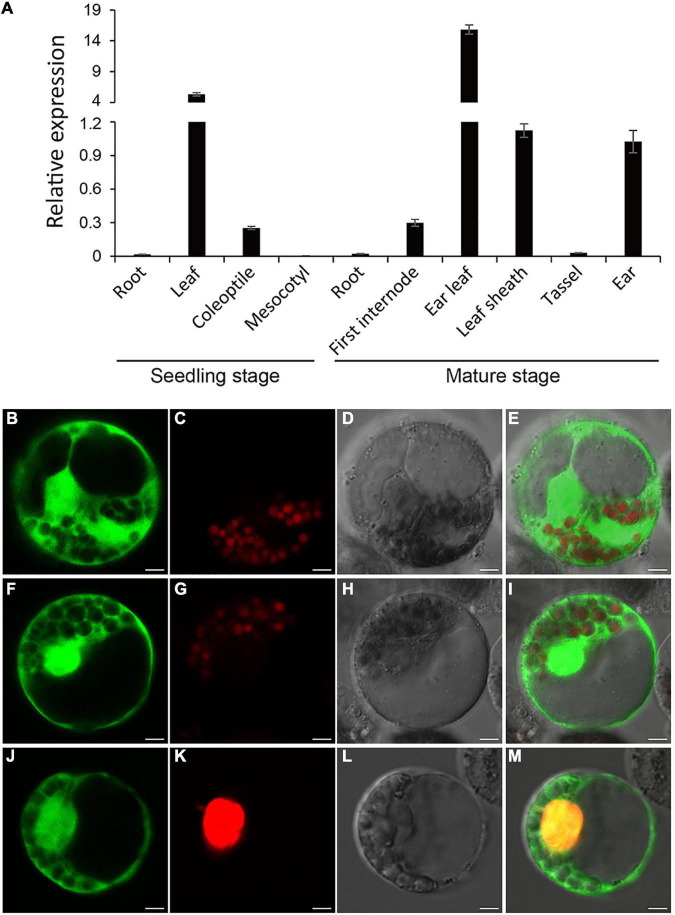
Expression pattern and of Subcellular localization of RppM. **(A)** Expression analysis of *RppM* in Jing2416K roots, leaves, coleoptiles, and mesocotyls at the seedling stage and roots, first internodes, leaf sheaths, tassels, and ears at the mature stage was performed by quantitative real-time PCR. Data are means ± SD of three biological replicates. **(B–E)** The free GFP. **(F–M)** Subcellular localization of RppM protein in maize protoplasts. **(F,J)** RppM-GFP fusion protein. **(C,G)** Chloroplast autofluorenscence. **(K)** The nucleus marker D53-mCherry. **(D,H,L)** Bright field image. **(E)** Merged image of **(B,C)**. **(I)** Merged image of **(F,G)**. **(M)** Merged image of **(F,G)**. Scale bar: 5 μm.

*RppM* basal expression in various organs of Jing2416K seedlings and mature plants was examined by quantitative real-time PCR (qRT-PCR). In seedlings, the strongest expression was detected in leaves; *RppM* was weakly expressed in coleoptiles, with scarcely any expression in roots or mesocotyls (Figure 3A). At the mature stage, *RppM* expression in ear leaves was approximately 10-fold higher than in leaf sheaths, ears, or the first above-ground internode, whereas barely any expression was detected in roots or tassels (Figure 3A). These results indicate that *RppM* was constitutively expressed in all organs and at all developmental stages examined, with the strongest expression detected in leaves at the mature stage, consistent with the main site of SCR infection.

To determine the subcellular location of RppM, the full-length CDS of RppM was fused to the *N*-terminus of green fluorescent protein (GFP). We transformed the plasmid containing the RppM-GFP fusion protein into maize protoplasts. The RppM-GFP protein was detected in both the nucleus and cytoplasm (Figures 3F–M). These results localized the RppM protein to the nucleus and cytoplasm.

### Southern Corn Rust Resistance Mechanism of Jing2416K Revealed by Transcriptome Analysis

To investigate the SCR resistance mechanism of Jing2416K, we performed transcriptome sequencing of Jing2416K and Jing2416 at two time points: 48 days after sowing (DAS), i.e., before the appearance of visible lesions in ear leaves of Jing2416, and 62 DAS, when lesions were clearly visible in ear leaves of Jing2416 (Supplementary Figure 4). Using the RNA-seq data, we carried out three sets of comparisons: Jing2416K vs. Jing2416 at 48 DAS (48 DAS_ J2416K vs. J2416), Jing2416K vs. Jing2416 at 62 DAS (62 DAS_ J2416K vs. J2416), and Jing2416K at 62 DAS vs. 48 DAS (J2416K_ 62 DAS vs. 48 DAS). To further explore the biological pathway possibly involved in the SCR resistance of Jing2416K, we identified “functional pathways” associated with differentially expressed genes (DEGs) based on KEGG and KOG databases.

In the 48 DAS_ J2416K vs. J2416 comparison, 314 DEGs were identified between Jing2416K and Jing2416, of which 198 were up-regulated and 116 were down-regulated (Supplementary Data 1). Up-regulated genes were associated with 31 functional pathways, including eight with more than two genes (Supplementary Figure 5 and Supplementary Data 1). A total of 88 DEGs between Jing2416K and Jing2416 were uncovered in the 62DAS_ J2416K vs. J2416 comparison, of which 51 were up-regulated and 37 were down-regulated (Supplementary Data 2). Eleven functional pathways were identified in up-regulated genes, including six with more than two genes (Supplementary Figure 6 and Supplementary Data 2). On both 48 DAS and 62 DAS, five functional pathways associated with up-regulated genes in Jing2416K were co-enriched: plant–pathogen interaction, cell wall component, plant hormone and signal transduction, cytochrome P450, and ubiquinone and other terpenoid-quinone biosynthesis.

**FIGURE 4:**
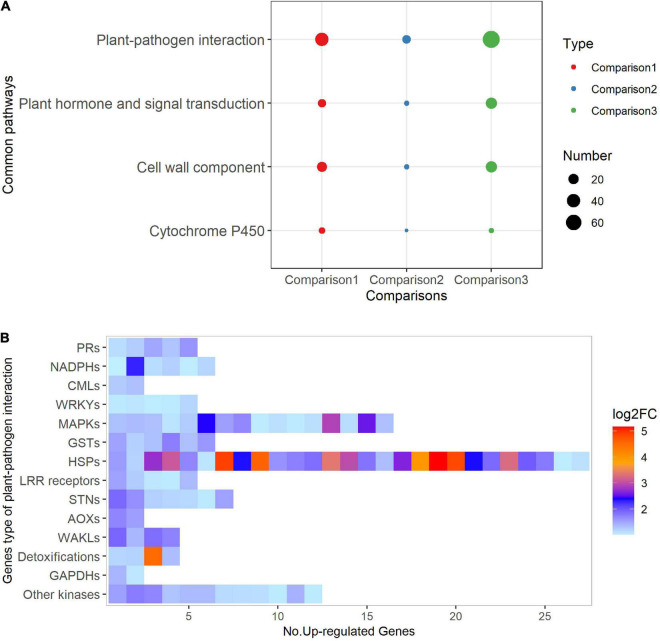
Functional pathways analysis of up-regulated genes. **(A)** Common pathways identified in up-regulated genes in all three transcriptome comparisons. Comparison1, 48 DAS_ J2416K vs. J2416; Comparison2, 62 DAS_ J2416K vs. J2416; Comparison3, J2416K_ 62 DAS vs. 48 DAS. **(B)** Heatmap of 14 types of genes associated with the plant–pathogen interaction pathway. PRs, pathogenesis-related genes; NADHs, NADPH oxidases; CMLs, CaM-like proteins; WRKYs, WRKY transcription factors; MAPKs, mitogen-activated protein kinases; GSTs, glutathione transferases; HSPs, heat shock proteins; STNs, serine/threonine protein kinases; AOXs, alternative oxidases; WAKLs, wall-associated receptor kinases; GAPDHs, glyceraldehyde-3-phosphate dehydrogenases.

We also identified 386 up-regulated genes (out of 589 DEGs) in J2416K_ 62DAS vs. 48DAS. Twenty-one of 43 up-regulated functional pathways were enriched in more than two genes, including 12 pathways associated with disease resistance responses (Supplementary Figure 7 and Supplementary Data 3). A total of four co-enriched pathways were identified in the three sets of comparisons, namely, plant–pathogen interaction, cell wall component, plant hormone and signal transduction, and cytochrome P450 (Figure 4A). Among these four pathways, plant–pathogen interaction accounted for most of the up-regulated genes participating in multiple signaling-related events in innate immunity, such as LRR receptors involved in recognition of pathogen-associated molecular patterns (PAMPs), MAPKs involved in phosphorylation of kinase cascades, CMLs involved in calcium signaling, WRKY transcription factors involved in regulation of defense-related genes, NADPH oxidases involved in ROS generation, and pathogenesis-related genes (Figure 4B). In addition, we identified 37 genes that were up-regulated in at least two comparisons (Supplementary Data 4). Taken together, these observations of functional pathway enrichment suggest that the immunity systems of Jing2416K were activated upon infection by the SCR pathogen.

### Development of Functional Kompetitive Allele-Specific PCR Markers for *RppM*

**FIGURE 5:**
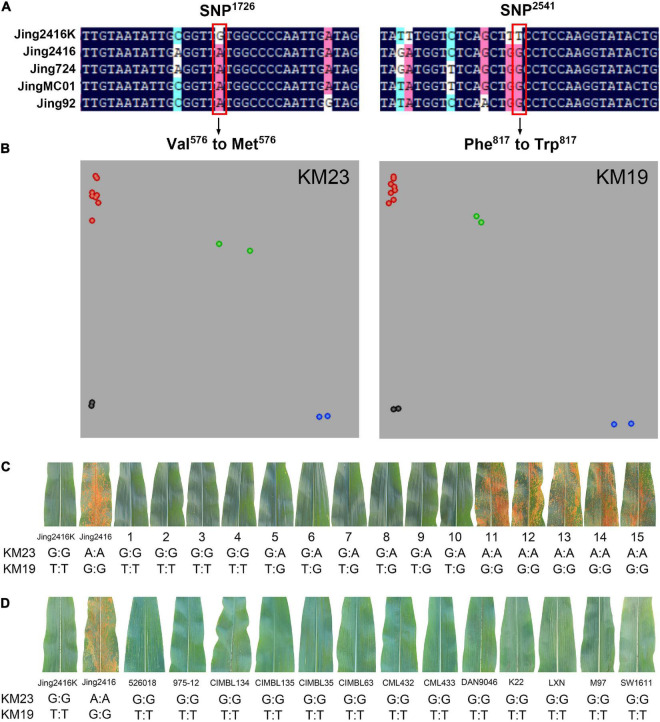
Development of Kompetitive allele-specific PCR (KASP) markers for *RppM* and genotype analysis. **(A)** The two conserved SNPs and changes of amino acids in resistant and susceptible lines. SNP sites are indicated by red boxes. **(B)** Genotyping of inbred lines Jing2416K, Jing2416, Jing724, JingMC01, and Jing92 and F_1_ plants of Jing2416 and Jing2416K with markers KM23 and KM19. Red, green, and blue dots represent susceptible, heterozygous resistant, and homozygous resistant genotypes, respectively. Black dots represent the non-template control (NTC). **(C)** Genotypes of F_2_ plants derived from Jing2416 and Jing2416K. 1–10, resistant plants; 11–15, susceptible plants. **(D)** Thirteen inbred lines of a GWAS population identified with markers KM23 and KM19, all exhibiting SCR resistance.

The development of specific molecular markers is required to facilitate the detection of the *RppM* gene and its application to breeding. We accordingly compared the CDS of the *RppM* allele among different inbred lines (resistant line Jing2416K and susceptible lines Jing2416, Jing724, JingMC01, and Jing92) and found two conserved single nucleotide polymorphisms (SNPs): SNP^1726^ and SNP^2451^. SNP^1726^ was a single-base substitution (G^1726^→7^1726^) in the second exon, resulting in the replacement of a Val residue by Met. SNP^2451^ was also a single-base substitution (T^2451^→4^2451^) in the second exon, but this change altered a Phe residue, leading to Trp (Figure 5A). We designed KASP markers KM23 and KM19 to detect SNP^1726^ and SNP^2451^, respectively, and performed genotyping of inbred lines Jing2416K, Jing2416, Jing724, JingMC01, and Jing92; F_1_ plants of Jing2416 and Jing2416K (Figure 5B); and F_2_ plants derived from Jing2416 and Jing2416K (10 resistant plants and 5 susceptible plants). Genotypes of the resistant plants were G:G and G:A for KM23 and T:T and T:G for KM19, whereas those of the susceptible plants were A:A (KM23) and G:G (KM19) (Figure 5C). We then used these two KASP markers to identify 527 inbred lines in a GWAS population (Yang et al., 2011), thereby obtaining 13 inbred lines with the resistant genotype that also exhibited resistance to SCR (Figure 5D). Genotypes of all individual plants were significantly correlated with SCR resistance phenotypes. Taken together, our results demonstrate that KM23 and KM19 can be used as functional allele-specific markers for *RppM*.

### Application of KM23 and KM19 in Marker-Assisted Selection

**FIGURE 6:**
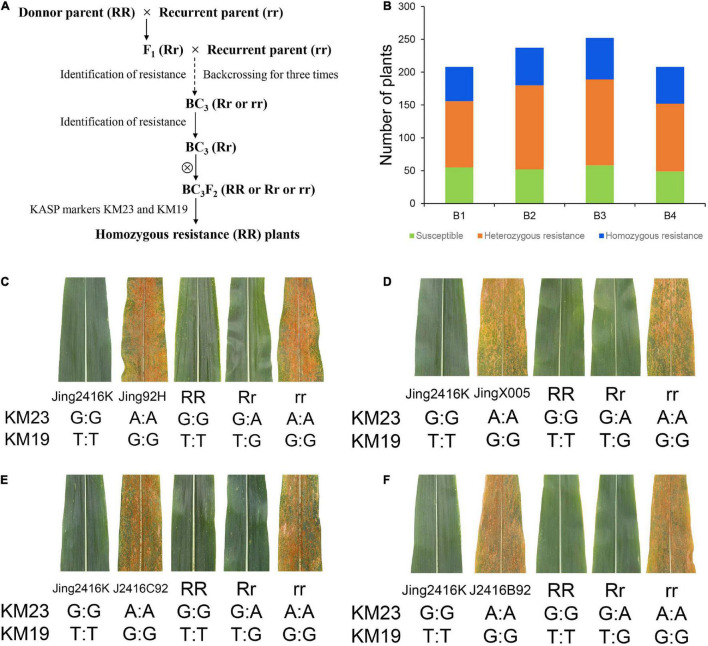
Application of KM23 and KM19 in marker-assisted selection. **(A)** Overview of the procedure used to generate homozygous lines resistant to SCR. **(B)** Number of plants of each genotype in the four BC_3_F_2_ populations B1, B2, B3, and B4 derived from crosses of Jing2416K with Jing92H, JingX005, Jing2416C92, and Jing2416B92, respectively. **(C–F)** Phenotypes and genotypes of BC_3_F_2_ individuals. RR, Rr, and rr respectively indicate homozygous resistant, heterozygous resistant, and susceptible plants in the four BC_3_F_2_ populations.

To further evaluate the applicability of KM23 and KM19 for MAS, we derived four BC_3_F_2_ populations from Jing2416K (donor parent) and susceptible lines Jing92H, JingX005, Jing2416C92, and Jing2416B92 (recurrent parent) via backcrossing (Figure 6A) and obtained 208, 237, 252, and 208 BC_3_F_2_ individuals, respectively. All BC_3_F_2_ plants were genotyped using the two KASP markers and were evaluated for SCR resistance, resulting in 52/57/63/56 homozygous resistant plants, 101/128/131/103 heterozygous resistant plants, and 55/52/58/49 susceptible plants, respectively (Figure 6B). Genotypes of all individual plants were significantly correlated with their SCR resistance phenotypes (Figures 6C–F). KM23 and KM19 can therefore be used as *RppM*-specific markers for MAS in populations derived from crosses between Jing2416K and susceptible inbred lines.

## Discussion

Because of global climate change, SCR has become a disastrous disease in major maize-producing regions of the world and now seriously threatens global maize production and food security (Mueller et al., 2020). Control of SCR in maize production is difficult given the wide dispersal of the anemochorous spores, rapid evolution, and dynamic population structure of this pathogen (Rochi et al., 2016; Gill et al., 2018). The identification of host *R* genes and the development of durable resistant inbred lines are considered to be the most effective, economical strategies to control SCR. A few inbred lines from tropical germplasm are resistant to SCR, the lack of effective resistance genes in temperate germplasm (Zhao et al., 2013). Thus, identification and isolation of novel *R* genes are urgently needed for maize breeding and germplasm improvement in temperate regions and thus enhance the SCR resistance of maize hybrids. In the present study, Jing2416K exhibited complete resistance to SCR over many years of cultivation and identification in field trials in Sanya. We confirmed that *ORF4* from Jing2416K is the functional *RppM* gene by overexpression analysis (Figure 2). The *RppM* gene encodes a typical CC-NBS-LRR protein localized in both the nucleus and cytoplasm (Figures 3F-M). The expression level of *RppM* was strongest in ear leaves at the mature stage; this suggests that *RppM*-mediated resistance occurs mainly in leaves of mature-stage maize (Figure 3A), consistent with the pattern of SCR-mainly occurs the leaves begins at the tasseling stage.

The most predominant *R* genes encode NBS-LRR proteins, which protect plants against various pathogens by recognizing different effectors. Examples include the Arabidopsis downy mildew resistance gene *RPP13* (Bittner-Eddy et al., 2001), the rice blast resistance gene *Pita* (Bryan et al., 2000), the barley powdery mildew resistance gene *Mla1* (Zhou et al., 2001), and the wheat leaf rust resistance gene *Lr10* (Loutre et al., 2009). In regard to SCR, previous studies have identified a number of major SCR resistance genes (Zhou et al., 2008; Yao et al., 2013; Zhao et al., 2013; Wu et al., 2015; Wang B. et al., 2019; Lv et al., 2020), but only *RppC* had been cloned. The *RppC* gene encodes an NLR-type immune receptor, which detects *Puccinia polysora* by recognizing the avirulence effector AvrRppC, in turn leading to effector-triggered immunity (Deng et al., 2022). Rather than even dispersal on chromosomes, *R* genes tend to be clustered in closely related gene groups (Meyers et al., 2005; Wang et al., 2007). Almost all SCR resistance genes currently identified, including *RppC* and *RppM*, are located on the short arm of chromosome 10. In our study, *RppM* was separated from *RppC* by a physical distance of approximately 1.1 Mb based on the B73 RefGen_v4 reference genome. This region of chromosome arm 10S may function as a vital genomic source of SCR resistance.

Plants use two different defense strategies to protect themselves from pathogen attack (Jones and Dangl, 2006; Zhang et al., 2012). The first strategy is PTI, which is mediated by pattern recognition receptors (PRRs) that recognize PAMPs and activate a downstream MAP kinase cascade. The second strategy is ETI, which occurs when plant R proteins sense specific pathogen effectors. Plant defense responses are usually accompanied by the synthesis and accumulation of phytohormones, such as jasmonic acid (JA), salicylic acid (SA), and ethylene (ET), which play an important role in the regulation of participating signaling pathways (Thaler et al., 2012; Louis et al., 2015; Yang et al., 2015). In addition, plant secondary metabolites, including lignin, zealexin, and kauralexins, are synthesized in response to pathogen infection, which results in the reinforcement of cell walls, an oxidative burst, and the accumulation of antimicrobial compounds against pathogens (Shirsekar et al., 2014; Yang et al., 2017; Fu et al., 2018). Cytochrome P450, which catalyzes an extremely diverse set of reactions, is involved in the biosynthesis or catabolism of plant secondary metabolites (Morant et al., 2003). In our study, functional pathway enrichment analysis suggested that the plant–pathogen interaction pathway was constitutively activated in Jing2416K (Supplementary Figure 8). Numerous genes involved in PTI were significantly up-regulated in Jing2416K, including pattern recognition receptors (e.g., *Zm00001d006117*, *Zm00001d002287*, and *Zm00001d003101*), MAPK kinases (e.g., *Zm00001d020100*, *Zm00001d025055*, and *Zm00001d047437*), calcium-dependent protein kinases (e.g., *Zm00001d028428*), WRKY transcription factors (e.g., *Zm00001d023336* and *Zm00001d039584*), and NADPH oxidase (e.g., *Zm00001d007411*, *Zm00001d038694*, and *Zm00001d040326*). A variety of genes involved in ETI were also significantly up-regulated in Jing2416K, such as heat shock proteins (e.g., *Zm00001d007271*, *Zm00001d008841*, and *Zm00001d012420*) and PRs (e.g., *Zm00001d008465* and *Zm00001d008468*). At the same time, hormone synthesis-related genes were significantly up-regulated in Jing2416K, including those related to SA (e.g., *Zm00001d032858*, *Zm00001d038087*, and *Zm00001d041082*), JA (e.g., *Zm00001d020614*, *Zm00001d027901*, and *Zm00001d047744*), and ET (e.g., *Zm00001d027686*), which suggests that phytohormones are involved in the regulation of defense responses in Jing2416K. Secondary metabolite biosynthetic genes were also significantly up-regulated in Jing2416K; examples include genes for MYB transcription factors (e.g., *Zm00001d010190* and *Zm00001d032240*) and cytochrome P450 subfamily members (e.g., *Zm00001d020340* and *Zm00001d013629*) as well as genes related to lignin synthesis (e.g., *Zm00001d021770*, *Zm00001d022457*, and *Zm00001d045092*), cellulase synthesis (e.g., *Zm00001d021818* and *Zm00001d023294*), and other phytoalexins (e.g., *Zm00001d011890*, *Zm00001d053436*, and *Zm00001d021358*). Collectively, both PTI- and ETI-related receptor genes as well as signaling molecules were significantly up-regulated in Jing2416K, thus suggesting that multiple defense systems were initiated in Jing2416K, including both PTI and ETI, reinforcement of cell walls, accumulation of antimicrobial compounds, and activation of phytohormone signaling pathways, thereby facilitating the complete resistance of Jing2416K to the SCR pathogen.

The development of specific markers for *R* genes and their application in MAS should significantly improve breeding efficiency and shorten the number of required breeding years. Many functional genes have been successfully deployed for the improvement of important traits and MAS in maize (Ning et al., 2011; Chai et al., 2012; Zhou et al., 2012; Zheng et al., 2014), but this has not been true to the same extent with SCR owing to the lack of *R* genes and corresponding specific markers. In our study, we accordingly compared the CDS of the *RppM* allele among resistant and susceptible inbred lines and developed 24 KASP markers using different SNPs (Supplementary Table 3). Genotypes of all tested individual plants were significantly correlated with their phenotypes by markers KM23 and KM19 (SNP^1726^ and SNP^2451^) but others cannot, which suggests that the two KASP markers can be used as functional allele-specific markers for *RppM* (Figures 5B–D). We then carried out SCR resistance improvement of four susceptible lines using *RppM* and the functional KASP markers and obtained homozygous resistant plants (Figure 6). Taken together, our observations indicate that the SCR resistance gene *RppM* and the functional KASP markers developed in this study have great potential utility in the cultivation of durable resistant maize cultivars.

## Data Availability Statement

The data presented in the study are deposited in the Sequence Read Archive (SRA) repository, accession number PRJNA849153.

## Author Contributions

JZ and WS designed the experiments. SW and XW performed the most of experiments and wrote the manuscript. RZ, QL, XS, and JW performed to identify the resistance and constructed population. YW, JX, YL, YZ, ZS, AS, CL, SX, YJ, ZL, and RW took part in the part of experiments and the manuscript modification. All authors contributed to the article and approved the submitted version.

## Conflict of Interest

The authors declare that the research was conducted in the absence of any commercial or financial relationships that could be construed as a potential conflict of interest.

## Publisher’s Note

All claims expressed in this article are solely those of the authors and do not necessarily represent those of their affiliated organizations, or those of the publisher, the editors and the reviewers. Any product that may be evaluated in this article, or claim that may be made by its manufacturer, is not guaranteed or endorsed by the publisher.

## Footnotes

1
https://web.expasy.org/compute_pi/

2
http://smart.embl-heidelberg.de/

3
http://cb.csail.mit.edu/cb/paircoil2/

4
https://meme-suite.org/meme/tools/meme

5
http://www.ncbi.nlm.nih.gov/

6
https://phytozome-next.jgi.doe.gov/

7
https://www.genscript.com/ssl-bin/app/primer

8
ftp://ftp.ncbi.nlm.nih.gov/blast/db/

9
http://web.expasy.org/docs/swiss-prot_guideline.html

10
http://pfam.xfam.org/

11
http://www.ncbi.nlm.nih.gov/COG/

12
http://www.geneontology.org

13
http://www.genome.jp/kegg/

14
https://probes.pw.usda.gov/batchprimer3/
