# Supplementary material for: RppM, Encoding a Typical CC-NBS-LRR Protein, Confers Resistance to Southern Corn Rust in Maize
Source: Front Plant Sci. 2022 Jul 12;13:951318. doi: 10.3389/fpls.2022.951318 (PMC9317930; doi:10.3389/fpls.2022.951318)
Supplement: Supplementary file 1 [file Data_Sheet_1.PDF]

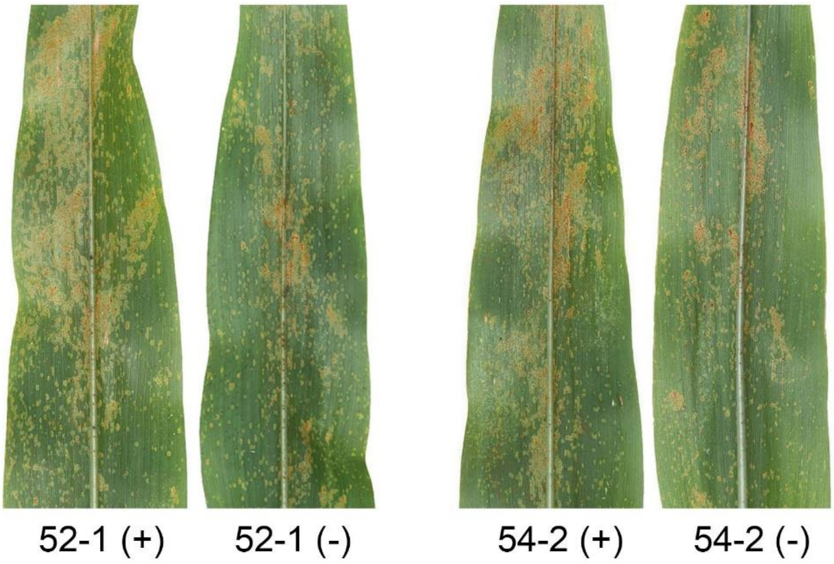

Supplementary Figure 1. Phenotypes of two independent T_1 overexpression lines (52-1 and 54-2) of *ORF6*.

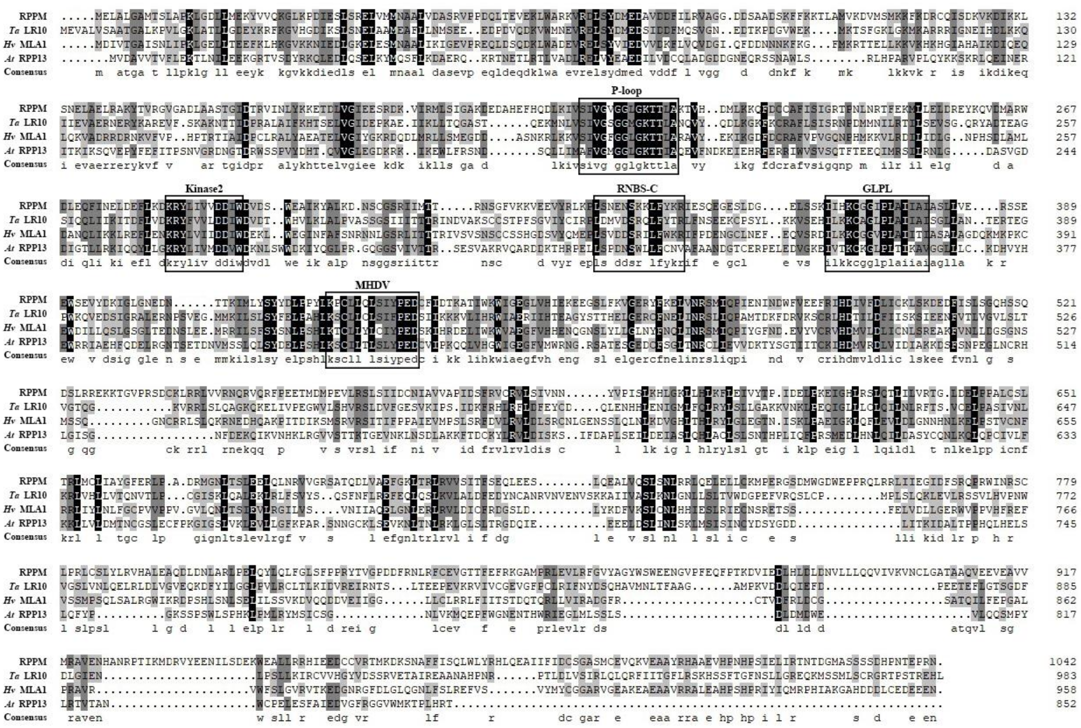

Supplementary Figure 2. Alignment of amino acid sequences of *RppM* with relevant CC-NBS-LRR proteins from other plant species.

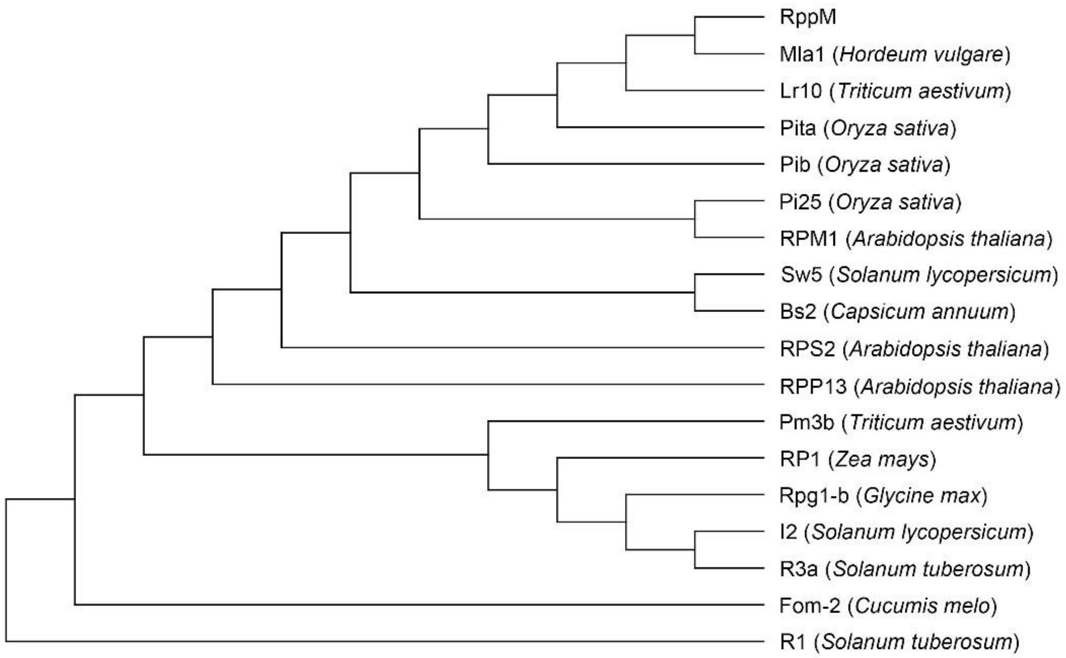

Supplementary Figure 3. Neighbor-joining phylogenetic tree of RppM from maize and relevant CC-NBS-LRR proteins from other plant species. The analyzed proteins confer resistance against the following pathogens in the indicated plant species: Arabidopsis RPM1 and RPS2, *Pseudomonas syringae*; Arabidopsis RPP13, *Hyaloperonospora parasitica*; rice Pita, Pib, and Pi25, *Magnaporthe oryzae*; maize RP1, *Puccinia sorghi*; wheat Lr10 and Pm3b, *Puccinia triticina* and *Blumeria graminis*, respectively; barley Mla1, *Blumeria graminis*; tomato Sw5 and I2, *Tospovirus* and *Fusarium oxysporum*, respectively; potato R3a and R1, *Phytophthora infestans*; pepper Bs2, *Xanthomonas campestris*; soybean Rpg1-b, *Pseudomonas syringae*; melon Fom-2, *Fusarium oxysporum*.

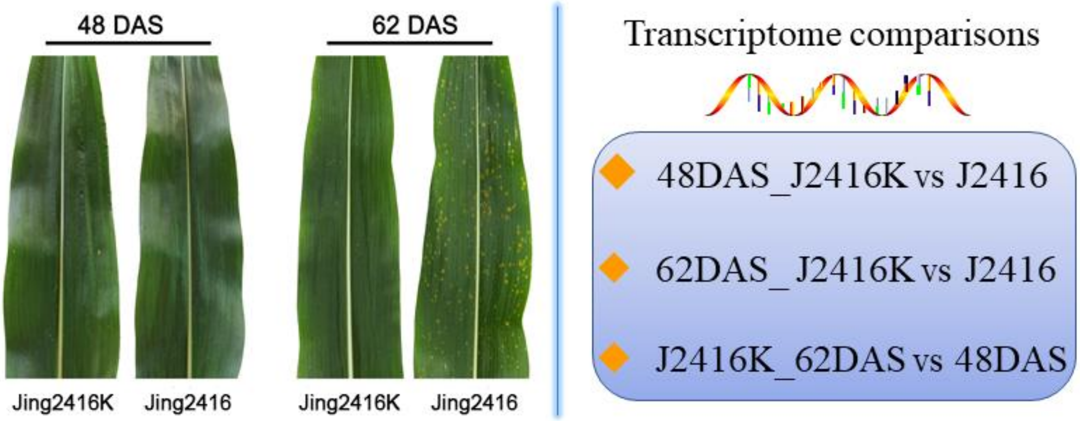

Supplementary Figure 4. Phenotypes of Jing2416K and Jing2416 at 48 and 62 DAS and comparison groups used in the transcriptome analysis.

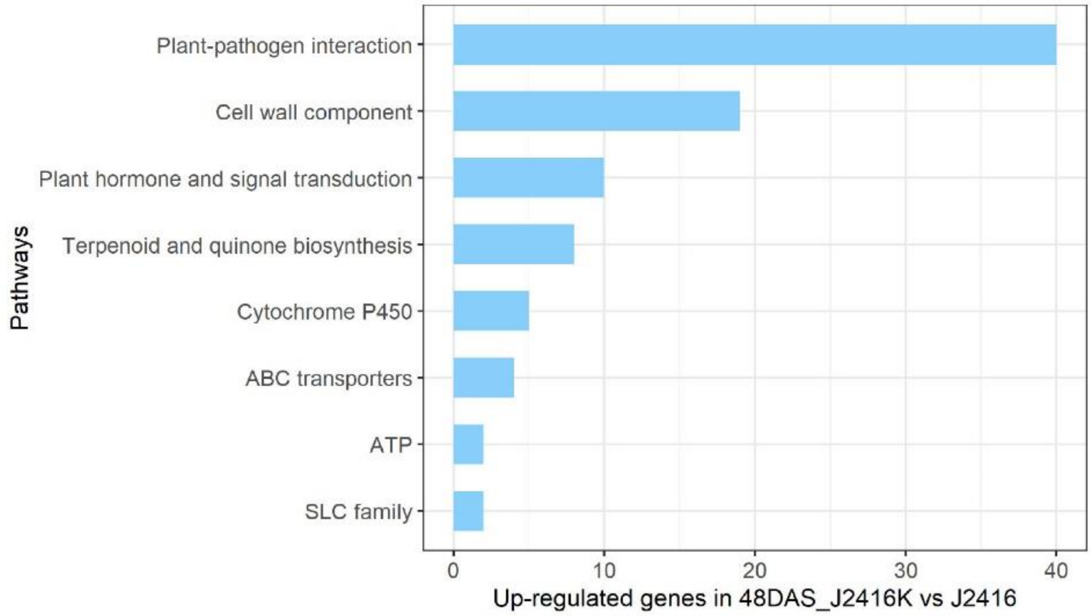

Supplementary Figure 5. Functional pathways identified in up-regulated genes between Jing2416K and Jing2416 at 48 DAS.

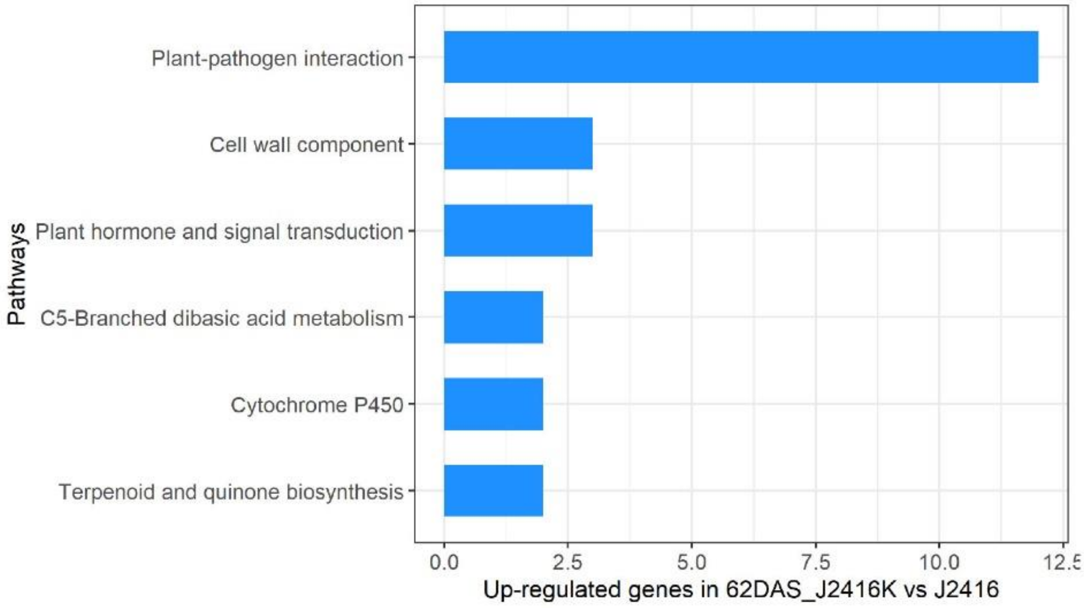

Supplementary Figure 6. Functional pathways identified in up-regulated genes between Jing2416K and Jing2416 at 62 DAS.

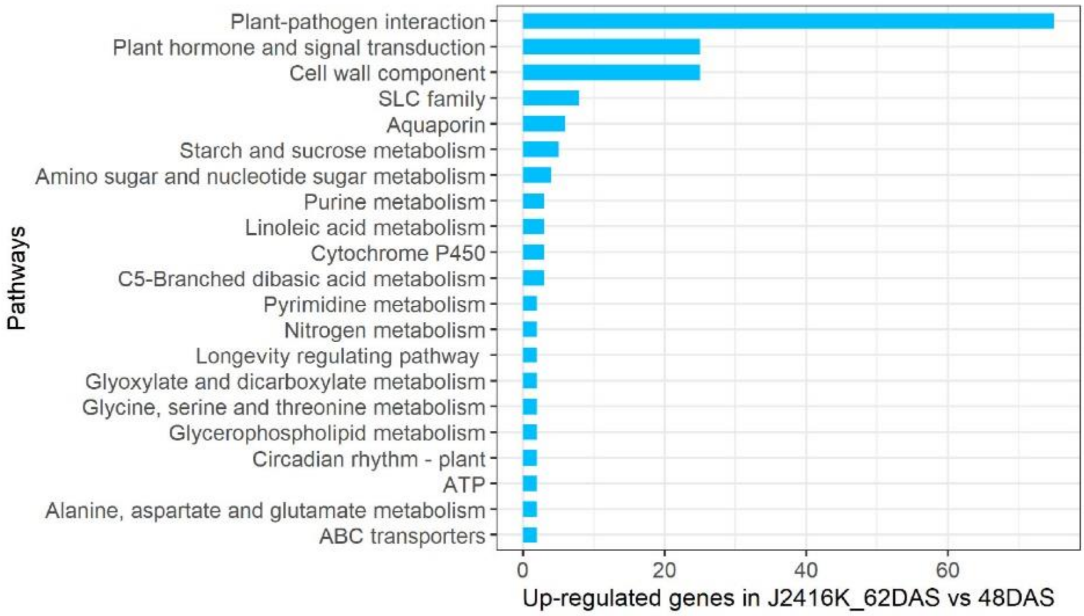

Supplementary Figure 7. Functional pathways identified in up-regulated genes in Jing2416K between 62 and 48 DAS.

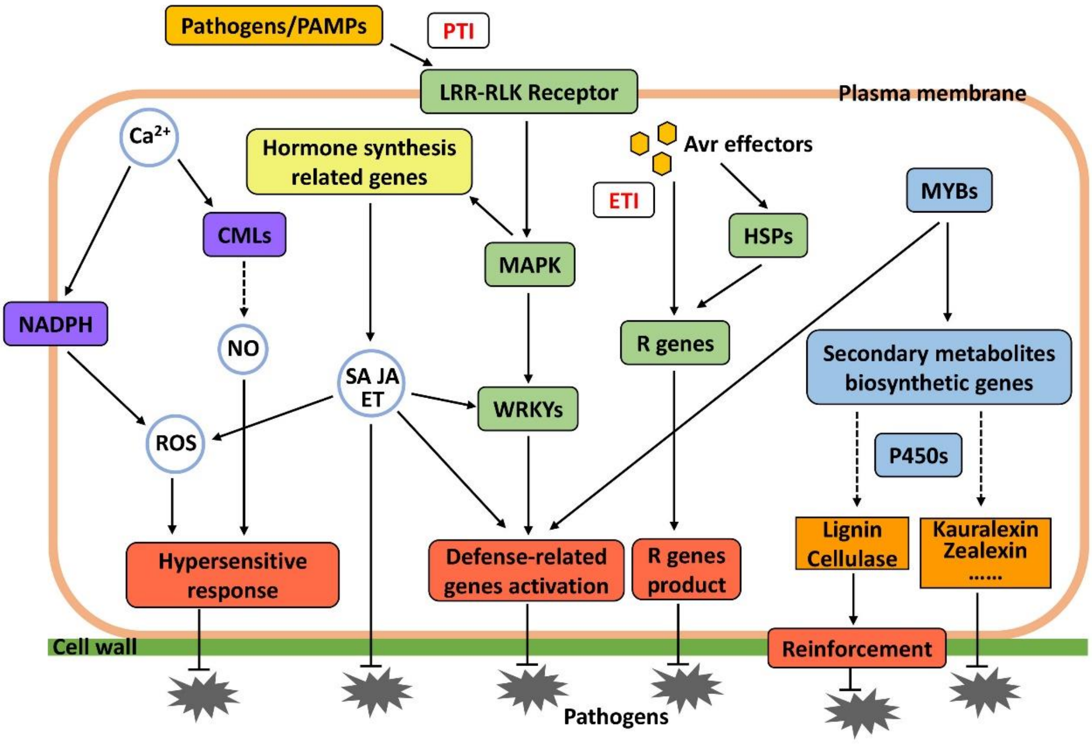

Supplementary Figure 8. Model of possible mechanisms involved in the resistance of Jing2416K to the SCR pathogen. The functions of participating genes are indicated as follows: green, PTI and ETI; violet, Ca^{2+} signaling pathway; yellow, hormone synthesis; and blue, secondary metabolite biosynthesis. The ensuing defense response events are shown in red. PAMPs, pathogen associated molecular patterns; PTI, PAMP-triggered immunity; ETI, effector-triggered immunity; LRR-RLK Receptor, leucine-rich-repeat receptor-like kinase; MAPK, mitogen-activated protein kinase; CMLs, CaM-like proteins; HSPs, heat shock proteins; MYBs, MYB transcription factors; WRKYs, WRKY transcription factors; R genes, resistance genes; NADPH, NADPH oxidase; P450s, cytochromes P450; SA, salicylic acid; JA, jasmonic acid; ET, ethylene; ROS, reactive oxygen species; NO, nitric oxide; Avr effectors, avirulence effectors.
