# Supplementary material for: RppM, Encoding a Typical CC-NBS-LRR Protein, Confers Resistance to Southern Corn Rust in Maize
Source: Front Plant Sci. 2022 Jul 12;13:951318. doi: 10.3389/fpls.2022.951318 (PMC9317930; doi:10.3389/fpls.2022.951318)
Supplement: Supplementary file 2 [file Data_Sheet_2.PDF]

**Supplementary Table 1.** Primers used in this study.

| Marker | Functions | Primer sequences（5' → 3'） | |
|---|---|---|---|
| | | Forward primer | Reverse primer |
| C65 | Construction of vector | TCGGGCTGCAGAATTCAGGTCGA GGTGGTGAGGT | CGAGAAGCTTGAATTCCATCACTT GGGTCCGTCT |
| C67 | Construction of vector | TCGGGCTGCAGAATTCAGGTGGT GAGGCATGGAGTT | CGAGAAGCTTGAATTCAAACCGAG TCATCAATTCCT |
| C65-GFP | Construction of vector | AGCAGATCTATCGATTCTAGAAT GGAGCTCGCCTTGGGG | TCCTTTGCCCATGGCTCTAGAATTC CTGGGCTCTGTGTT |
| G65-1 | Sequencing of *RppM* | AGGTCGAGGTGGTGAGGT | TATTGTCCTTTAAGGCATATTTGAT |
| G65-2 | Sequencing of *RppM* | GACTGTTGTGCTTTTATTTCTA | GATGCTTTAGGCTGATGGGTA |
| G65-3 | Sequencing of *RppM* | TAAGTTGTCCAAGGATGAAG | AGTTGACTTTGACTATTACTTGCT |
| G65-4 | Sequencing of *RppM* | TAGAGCTTTTGTGTAAAATGCCA | CATCACTTGGGTCCGTCT |
| Actin | RT-PCR | TCCATCATGAAGTGCGACGT | AGTAATCTCCTTGCTCATGC |
| RT65 | RT-PCR | AATGGTGTGCCGTTCGAG | AGTTGACTTTGACTATTACTTGCT |

**Supplementary Table 2.** Primer sequence of KASP markers KM23 and KM19.

| Marker | Primer sequences（5' → 3'） | | |
|---|---|---|---|
| | Primer_AlleleFAM | Primer_AlleleHEX | Primer_Common |
| KM23 | GAAGGTGACCAAGTTCATGCTCT GAAGCTAYCAATTGGGGCCAC | GAAGGTCGGAGTCAACGGATTC TGAAGCTAYCAATTGGGGCCAT | GCAGCGCTTCCC TGAAGAAACCAT |
| KM19 | GAAGGTGACCAAGTTCATGCTGC CAACAGTATACCTTGGAGGA | GAAGGTCGGAGTCAACGGATTG CCAACAGTATACCTTGGAGGC | ACAGGACCTAGA TAATCTAGCG |

**Supplementary Table 3.** The position and SNPs of 24 KASP markers.

| Markers | SNP_ID | allele_X | allele_Y | Markers | SNP_ID | allele_X | allele_Y |
|---|---|---|---|---|---|---|---|
| KM1 | 45 | T | A | KM13 | 1623 | T | G |
| KM2 | 64 | A | G | KM14 | 1942 | C | G |
| KM3 | 358 | T | C | KM15 | 2039 | A | G |
| KM4 | 522 | T | C | KM16 | 2075 | A | C |
| KM5 | 818 | T | A | KM17 | 2089 | T | C |
| KM6 | 818 | A | T | KM18 | 2310 | G | A |
| KM7 | 1030 | C | T | KM19 | 2451 | T | G |
| KM8 | 1162 | A | C | KM20 | 2650 | C | G |
| KM9 | 1209 | G | A | KM21 | 698 | G | C |
| KM10 | 1360 | A | C | KM22 | 1628 | T | C |
| KM11 | 1472 | A | G | KM23 | 1726 | G | A |
| KM12 | 1538 | G | C | KM24 | 1983 | C | G |
